# The More, The Better: “Do the Right Thing” For Natural Killer Immunotherapy in Acute Myeloid Leukemia

**DOI:** 10.3389/fimmu.2017.01330

**Published:** 2017-10-19

**Authors:** Sarah Parisi, Mariangela Lecciso, Darina Ocadlikova, Valentina Salvestrini, Marilena Ciciarello, Dorian Forte, Giulia Corradi, Michele Cavo, Antonio Curti

**Affiliations:** ^1^Department of Experimental, Diagnostic and Specialty Medicine, Institute of Hematology L. and A. Seràgnoli, S. Orsola-Malpighi Hospital, University of Bologna, Bologna, Italy

**Keywords:** natural killer cells, acute myeloid leukemia, immunotherapy, biomarkers, cell dose

## Abstract

Natural killer (NK) cells are circulating CD3^−^ lymphocytes, which express CD56 or CD16 and an array of inhibitory receptors, called killer-immunoglobulin-like receptors (KIRs). Alloreactive KIR-ligand mismatched NK cells crucially mediate the innate immune response and have a well-recognized antitumor activity. Adoptive immunotherapy with alloreactive NK cells determined promising clinical results in terms of response in acute myeloid leukemia (AML) patients and several data demonstrated that response can be influenced by the composition of NK graft. Several data show that there is a correlation between NK alloreactivity and clinical outcome: in a cohort of AML patients who received NK infusion with active disease, more alloreactive NK cell clones were found in the donor repertoire of responders than in non-responders. These findings demonstrate that the frequency of alloreactive NK cell clones influence clinical response in AML patients undergoing NK cell immunotherapy. In this work, we will review the most recent preclinical and clinical data about the impact of alloreactive NK cells features other than frequency of alloreactive clones and cytokine network status on their anti-leukemic activity. A better knowledge of these aspects is critical to maximize the effects of this therapy in AML patients.

## Introduction

In the last years, major advances have been achieved in the understanding of acute myeloid leukemia (AML) biology (1). However, these preclinical results have weakly impacted on the clinical outcome of AML patients, whose prognosis is overall largely unsatisfactory (1–4). A significant improvement in AML outcome is provided by allogeneic stem cell transplantation (SCT), where alloreactive T cells and natural killer (NK) cells mediate the graft-versus-leukemia (GvL) effect, contributing to leukemia eradication and relapse prevention. However, for many patients, the cellular immunotherapy within SCT is hampered by the high risk of treatment-related morbidity and mortality. To expand the option of cellular immunotherapy besides the SCT setting, adoptive transfer of effector immune cells is a promising approach (5, 6).

Natural killer cells are circulating CD3^−^ lymphocytes, which express CD56 or CD16. They account for 5–15% of circulating lymphocytes in humans and can be categorized into subpopulations with different maturation status and functional specificities. CD56^lo^CD16^+^ NK cells with high cytotoxic potential are predominant in human blood, while the immunomodulatory CD56^hi^CD16^−^ subset is more predominant in lymph nodes (7). NK cells mediate innate immune response and cancer immunological control independently from major histocompatibility complex (MHC)-based antigen recognition (8). A net effect between inhibitory receptors, such as killer-Immunoglobulin-like receptors (KIRs) and C-type lectin receptor (NKG2A), and activating receptors, including natural cytotoxic receptors (NCR), NKp46, NKp30, NKp44, and NKG2D, drives NK cell cytotoxic activity (9–11). Inhibitory KIRs are the most clinically relevant and interact with self and not self-HLA class I ligands (HLA-Bw4, HLA-C1, and HLA-C2) (12). During their maturation process, NK cells acquire cytolytic activity by upregulating cytotoxic receptors and expressing perforin under the stimulation of the T-cell derived cytokine interleukin (IL)-2. The subsequent “licensing” or “education” process is crucial for acquiring self-tolerance, avoiding NK-mediated self-damage (13). Several cytokines, such as interferon (IFN)-γ, tumor necrosis factor (TNF)-α, granulocyte-macrophage colony-stimulating factor (GM-CSF), and chemokines, such as CCL3, CCL4, and CCL5, are produced by stimulated NK cells, which, then, acquire the capacity of killing target cells, including tumor cells, through the perforin/granzyme pathway (14). As for AML, the first evidence of NK-mediated anti-leukemia effect was reported in the haploidentical T-cell depleted SCT setting, where it was demonstrated that a KIR-ligand mismatch between donor and recipient protects patients from leukemia relapse (15, 16).

## NK Immunotherapy for AML: The State-of-Art of Clinical Trials

Acute myeloid leukemia cells are more sensitive to NK-mediated cytotoxicity than solid tumors (7). For this reason, several groups have been prompted to exploit in AML the potential of NK cell adoptive immunotherapy (17). High risk patients with myeloid malignancies were safely infused with highly purified NK cells to consolidate engraftment after haploidentical SCT (18), thus providing the rationale for exploring this strategy as a means of increasing GvL without concurrent GVHD effects (19). Besides the transplantation setting, adoptive immunotherapy with haploidentical NK cells in AML was exploited in adults and in childhood patients (20–23). Altogether, these studies demonstrated that NK cell-based adoptive immunotherapy is safe and feasible in AML patients. Of note, donor NK cells expanded after infusion and were *in vivo* alloreactive against recipient’s leukemic cells with some promising clinical responses. To increase the number of NK cells to be infused, *ex vivo* NK cell expansion is under preclinical and early clinical investigation. Co-culture of NK cells with feeder cells plus cytokines is effective in generating large number of NK cells with high antitumor effect and long survival (24). Clinically, expanded NK cells have proven to be safe and feasible (25). Very recently, a first-in-man study investigated the role of memory-like NK cells, obtained after *in vitro* differentiation from human NK cells with IL-12, IL-15, and IL-18 (26).

## In Search for Biomarkers Predictive of Response to NK Immunotherapy for AML

**Figure 1:**
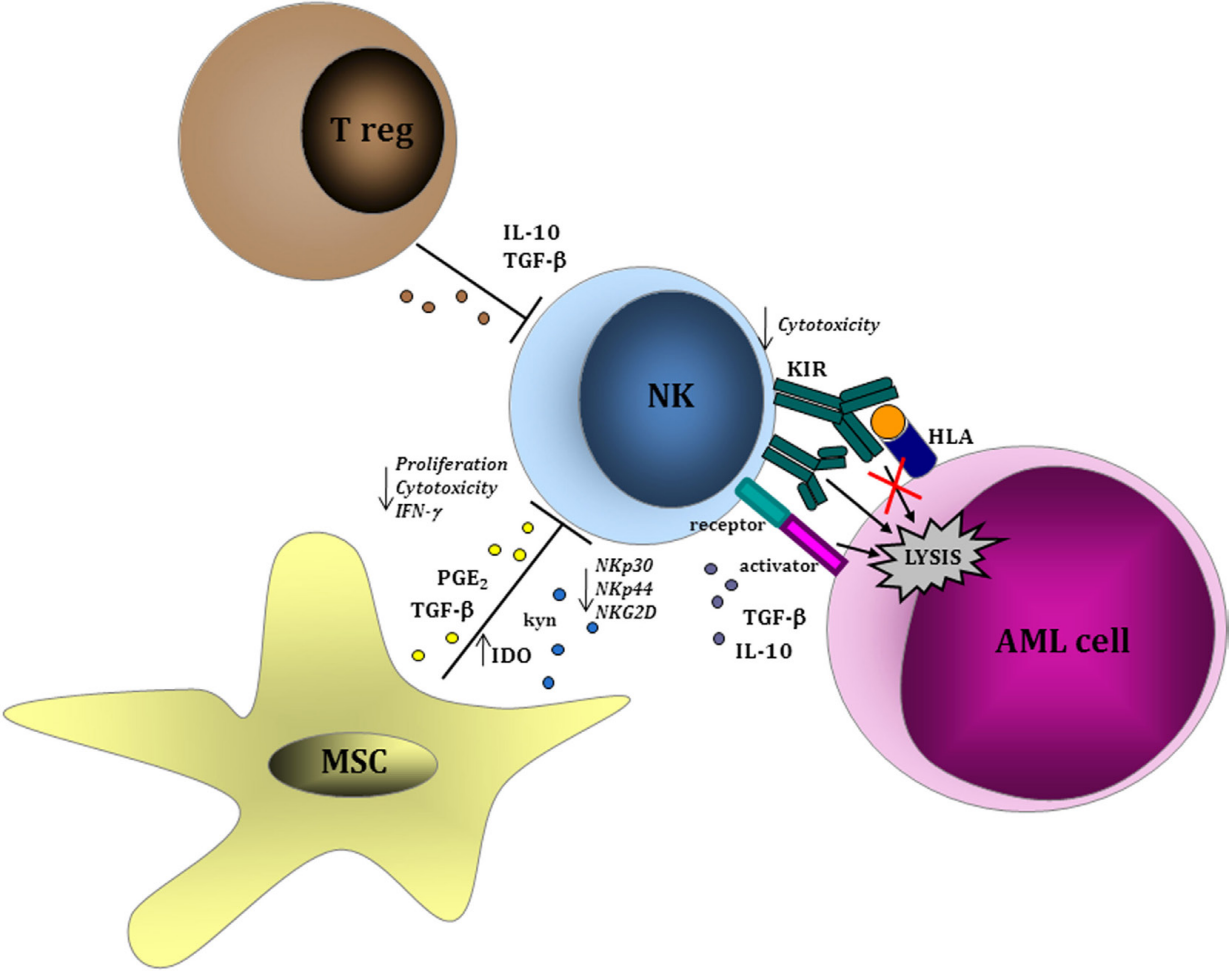
Leukemia-cell intrinsic and extrinsic factors influencing natural killer (NK) cell function. NK cells are able to recognize acute myeloid leukemia (AML) blasts in a major histocompatibility complex-unrestricted manner inducing cell lysis through perforin/granzyme pathway. Within tumor microenvironment, regulatory T lymphocytes and MSCs play a role in inhibiting NK cell-mediated proliferation and cytotoxic function *via* the production of soluble factors, such as interleukin (IL)-10, transforming growth factor (TGF)-β, and indoleamine 2,3-dioxygenase (IDO), thus resulting in the downregulation of activating receptors (NKp30, NK944, and NKG2D) and in the upregulation of inhibitory receptors. Similar evasion mechanisms have been described as a consequence of a direct effect from AML cells on NK cells.

Within clinical trials, “biology-driven approaches” have the potential to identify an array of biomarkers, which may be used to predict clinical response to NK immunotherapy and/or to guide clinical decision process. Some of these biomarkers may derive from the donor repertoire, whereas some others are related to host modifications after NK cell infusion (Figure 1).

### KIR–KIR-L Mismatch

The NK cells cytolytic activity is regulated by inhibitory KIRs, which mediate self-tolerance by engaging HLA class I antigens. Four different inhibitory KIRs seem to be primarily involved in inducing NK alloreactivity because of a stronger recognition of HLA class I ligands: KIR2DL2/2DL3 recognize HLA-C1 ligands (HLA-Cw), KIR3DL1 recognizes Bw4 haplotypes, and KIR3DL2 targets HLA-A3 and HLA-A11 ligands. NK alloreactivity can be predicted according to four models. The “missing-self” or “KIR-ligand mismatch” or “ligand incompability model” is based on the absence in the recipient of inhibitory HLA-I molecules that are present in the donor. According to the “receptor–ligand” model, donor NK cells are activated through inhibitory KIRs, which do not bind their HLA ligands in the recipient. The “missing-ligand” model predicts alloreactivity when at least one HLA-ligand is missing. The last of the models mentioned above states that activating KIRs on donor cells are needed in order to achieve NK cell alloreactivity (27). In haploidentical SCT, KIR-L mismatch dramatically impacts on the efficacy of NK cells in enhancing anti-leukemia effect (15, 16, 23). Whether these findings may be directly translated into the immunotherapy field has not been fully elucidated. In their pioneering study of adoptive NK immunotherapy, Miller et al. showed that KIR-L mismatch between donor and recipient had a predictive value in terms of clinical response (20). These data have not been confirmed in the subsequent study on a wider number of patients (28). Moreover, KIR-L mismatch between recipient and donor was not enough to translate into a significant clinical benefit for previously selected KIR-L-mismatched donor–recipient pairs (20). These results may indicate that a deeper characterization of KIR–KIR-L interaction is probably needed in the setting of NK immunotherapy. Donors with group B KIR haplotypes have been shown to improve survival of AML patients undergoing unrelated SCT (29). Very recently, based on specific HLA/KIR subtype combinations, a predictive algorithm for donor selection has been developed in a cohort of 1,328 patients with AML who received HLA-matched SCT transplant (30). Then, the selection of donors with favorable KIR genes of the B haplotype and/or with specific HLA/KIR subtype combinations may be used to increase the potential benefit of KIR–KIR-L mismatch between donor and recipient.

### Host Regulatory T Cells

Regulatory T lymphocytes (Tregs) are distinct CD4, CD25, and Foxp3 positive immunosuppressive lymphocytes, which induce tolerance by inhibiting immune responses, including NK cell-mediated cytotoxicity. During the development of malignancies, Tregs proliferate under the stimulation of transforming growth factor (TGF)-β secreted by myeloid dendritic cells (DCs) and accumulate in blood, draining lymph nodes, and tumor microenvironment (14). Many studies demonstrated that Tregs are able to influence NK cell activity (14, 28, 31). In particular, NK cell inhibition induced by Tregs is mediated by soluble and Tregs-bound TGF-β, both resulting in downregulating of NKG2D on NK cells (14). Accordingly, in mice Treg depletion with anti-CD25 monoclonal antibody abrogated tumor growth and allowed the proliferation of NK cells (32). The influence of Tregs in NK adoptive immunotherapy has been recently addressed by Miller et al. in a large group of patients infused with NK cells. In this study, 57 patients with relapsed or refractory AML who received lymphodepleting chemotherapy with fludarabine and cyclophosphamide followed by NK cell infusion were analyzed. Patients were divided into three subgroups on the basis of the methods of NK cell product preparation: cohort 1 received NK cells after CD3 depletion alone, cohort 2 received NK cells after CD3 depletion followed by CD56 positive selection, and cohort 3 received NK cells after a single-step CD3/CD19 depletion. Patients included in Cohort 3 received the highest NK cell dose and after NK infusion were treated with IL-2 diphteria toxin (IL-2DT), a cytotoxic fusion protein able to selectively deplete high affinity IL-2 receptors (CD25)-expressing cells, including Tregs. A clear association between NK expansion and clinical responses was documented and such correlation was partly dependent on host Tregs. These data suggest that Tregs frequency in patients infused with NK cells may be a biomarker predictive of NK cell expansion and activity and may have clinical implications for the choice and schedule of cytokine-based therapy after NK cell infusion. In particular, the potent effect of IL-2, commonly used in NK-based immunotherapy protocols, on NK cell expansion and activity may be counterbalanced by its detrimental proliferative activity on Tregs. Clinical trials, including post-infusion administration of cytokines, such as IL-15, with no proliferative effect on Tregs are highly warranted.

### Leukemia-Cell Intrinsic and Extrinsic Factors

A large body of evidence support the interplay between AML cells and the different cell components of the immune system (33). Some reports indicate that NK cells may be defective in AML patients (34, 35). In particular, a deficient expression of NCRs in NK cells from AML patients and during leukemia evolution has been observed (36). Conversely, NK cells with an activated phenotype from AML patients are cytotoxic against primary AML cells (37). Leukemia-induced phenotypic and functional NK cell abnormalities have been described in AML patients and are predictive of remission-induction after chemotherapy (38). Soluble factors, including IL-10, TGF-β, or indoleamine 2,3-dioxygenase (IDO), have been correlated with AML-mediated impairment of NK function (39). Sera collected from human AML patients contain microvescicles enriched in TGF-β, which mediates the inhibition of NK cell activity (40). In support of a pathogenetic role of these mechanisms during AML development, the defects of NK cells were restored in patients achieving complete remission after chemotherapy (38, 40). Moreover, the expression by AML cells of the immunosuppressive surface proteins CD200 (41) and CD137 (42) has a direct suppressive effect on NK cell activity, thus contributing to leukemia escape from NK-mediated control. The role of leukemic bone marrow (BM) microenvironment is critical in mediating immunotolerance (43–45). An increasing body of evidence has shown that immune effector cells are dysfunctional as a consequence of inhibitory signals deriving from a wide variety of immune cell components of tumor microenvironment, such as tolerogenic DCs, macrophages, and myeloid-derived suppressor cells (MDSCs). In solid tumors, these immunosuppressive cell subsets are expanded in patients and their presence in the tumor microenvironment correlates with worse prognosis and a dysfunctional activity of NK cells (46–48). In AML, the role of these immunosuppressive cells is not well-understood yet, whereas the activity of mesenchymal stromal cells (MSCs) on NK cells in BM leukemic microenvironment has been widely investigated. In particular, MSCs can negatively interfere with the function of NK cells by reducing their cytotoxic activity and cytokine production (49). Similar to the effects derived from AML cells, MSC-driven impairment of NK cell function was related to a downregulation of the activating NK receptors NKp30, NKp44, and NKG2D and mediated by IDO and prostaglandin E_2_. Whether these evasion mechanisms by AML cells and by other cell components of leukemic BM niche may be active during adoptive NK cell transfer has not been specifically addressed within clinical trials. Future immunotherapy trials are expected to take into accounts some of these parameters, which may be correlated with response to NK immunotherapy.

## NK Immunotherapy in AML: The Question of Dose

In SCT and cell therapy, how many cells should be infused to achieve a therapeutic effect represents a common and crucial question. Many efforts have been made, for example, to identify the minimum and optimal number of hematopoietic CD34^+^ stem/progenitor cells capable of a durable engraftment in autologous SCT (50) or, in the setting of allogeneic SCT, the dose of CD3^+^ T cells to be used as donor lymphocytes infusions (DLI) for relapsing leukemia (51, 52). For NK immunotherapy, whether the dose of infused NK cells really impacts on the clinical response is still a matter of debate. Although infused NK cells may eradicate AML cells in virtue of several factors other than their dose, preclinical studies have demonstrated that NK cell cytotoxicity is directly correlated to the ratio between NK and target cells (53–55). Then, to infuse an adequate number of NK cells is likely to be clinically relevant. Since NK cells account for only a small portion of circulating lymphocytes, several groups are exploring the option of increasing the number of infused NK cells by *ex vivo* expanding cytotoxic NK cells and different NK cell production protocols are under investigation (56). An overview of these strategies is out of the focus of the current review. Indeed, we consider a preliminary and fundamental step to address the question of the minimum number of effective NK cells to be infused as a means of adoptive immunotherapy. Under this viewpoint, there is no consensus on the parameters to be used for enumerating functionally active NK cells among infused cells, which increases variability among clinical studies and makes the comparison of clinical results extremely difficult, if not impossible (57). In a pediatric cohort of AML patients undergoing NK cell infusion as part of their post-remission strategy, mainly because of body weight discrepancy between donor and recipient, high numbers of total CD56^+^CD3^−^ NK cells/kg were infused (21). Of note, CD56^+^CD3^−^ NK cells were highly purified and the amount of contaminating cells, other than NK cells, was minimal. Interestingly, the clinical results were remarkable with 100% of patients achieving durable response. In our recent report on a group of elderly AML patients, who were infused with the same highly purified NK cell population as the pediatric study, a trend for better outcome in those patients who received higher numbers of NK cells was shown (23). In the largest study by Miller et al. (28), comparing the clinical results of 57 patients undergoing infusion of NK cells, total NK cell dose resulted as being not correlated with clinical response. Notably, in this study (28), three different methods for NK cell manufacturing were used and only in a minority of patients NK cells were highly purified as the studies above. Moreover, cell dose was not correlated with *in vivo* expansion and clinical response when purified NK cells were *in vitro* primed with IL-12, IL-15, and IL-18 (26). Although not conclusive, the results from these studies may suggest that the optimal NK cell dose may differ depending on the cell manufacturing system and that, in case CD56^+^CD3^−^ NK cells are highly purified and not manipulated, the simple count of total infused NK cells may be used as a useful parameter to predict clinical response.

**Figure 2:**
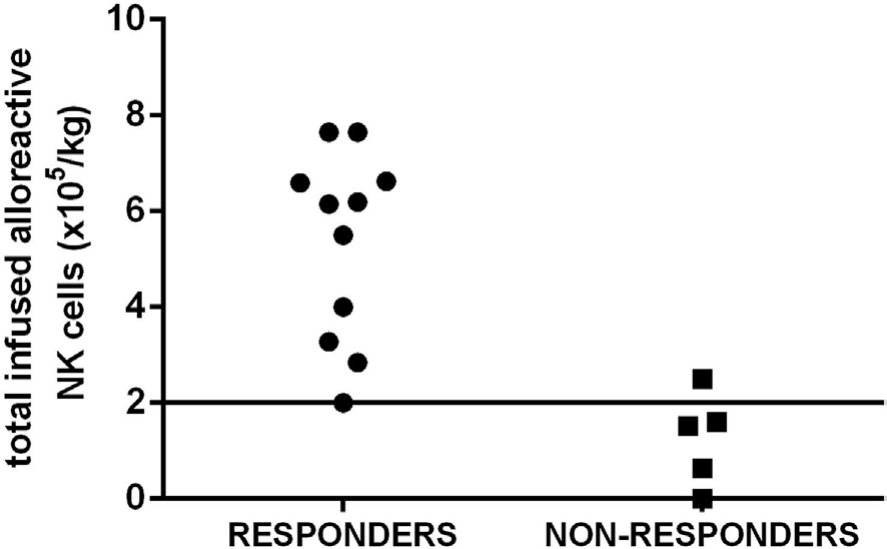
The “functional dose” of infused alloreactive natural killer (NK) cells is highly predictive of response in acute myeloid leukemia (AML) patients. A cohort of elderly AML patients in first complete remission received highly purified NK cells after lymphodepleting fludarabine and cyclophosphamide to prevent disease relapse. A better response in leukemia control was observed when at least 2 × 10^5^ functionally alloreactive donor NK cells/kg were infused. Such threshold has been chosen as a “functional cell dose” to be used to select donor(s) and guide NK cell processing.

The possibility of infusing a number of functionally active NK cells is likely to have greater impact on the efficacy of NK infusion than simply enumerating the number of total NK cells. Indeed, in our cohort of elderly AML patients, a better response was observed when at least 2 × 10^5^ functionally alloreactive donor NK cells/kg were infused (Figure 2). Our results indicate a highly predictive threshold of NK cells to be defined at the functional level (“functional dose”). Although such dose may be used to guide NK cell processing, the feasibility of such approach, which is based on a laborious and time-consuming alloreactive NK cell cloning, represents a major concern. Then, a major achievement will be to identify a functional dose of NK cells, predictive of response to NK cell infusion, by using alternative methods and tools. By moving from the results in the transplant setting (17), a new and predictive-of-response expression platform to be used in donor selection is likely to be a concrete possibility. The development of novel anti-KIR monoclonal antibodies that can distinguish inhibitory versus activating KIRs allows to identify alloreactive NK subpopulations in the majority of individuals at the phenotypical level. Although these new methods and tools require formal clinical testing and validation in large cohorts of patients undergoing NK immunotherapy, we may expect that the “functional dose” of NK cells to be infused will be identified without standard and cumbersome functional studies of NK cell cloning (58–60). Such approach will, then, allow to dissect donor NK cell repertoire at baseline, thus leading to definitively address the question of the correlation between NK cell dose and clinical response.

## Conclusion

Although several clinical reports have clearly paved the way for exploiting NK cell-based adoptive immunotherapy for AML patients, several crucial issues remain to be established and settled. In particular, the founding question of the therapeutic dose of infused NK cells potentially exerting *in vivo* anti-leukemia effect is still unanswered. Preliminary data from recent studies have shed light on this point, although the great heterogeneity of clinical strategies, which include freshly isolated *versu*s expanded, non-manipulated *versus* cytokine-primed NK cells, makes the comparison among trials extremely difficult. Advancements of diagnostic tools, coupled with a “biology-driven” approach, are expected to provide an array of biomarkers, which may help in selecting the best donor for the best patient, thus making NK immunotherapy really beneficial for a specific subset of AML patients.

## Author Contributions

SP wrote and revised the final manuscript; ML and DO provided figures and revised the final manuscript; VS, MC, DF, and GC contributed to manuscript preparation and editing; AC contributed to manuscript writing and final revision.

## Conflict of Interest Statement

The authors declare that the research was conducted in the absence of any commercial or financial relationships that could be construed as a potential conflict of interest.

## References

[1] Döhner H Estey E Grimwade D Amadori S Appelbaum FR Büchner T Diagnosis and management of AML in adults: 2017 ELN recommendations from an international expert panel. Blood (2017) 129:424–47. 10.1182/blood-2016-08-733196 27895058 PMC5291965

[2] Döhner H Weisdorf DJ Bloomfield CD Acute myeloid leukemia. N Engl J Med (2015) 373:1136–52. 10.1056/NEJMra1406184 26376137

[3] Bullinger L Döhner K Döhner H Genomics of acute myeloid leukemia diagnosis and pathways. J Clin Oncol (2017) 35:934–46. 10.1200/JCO.2016.71.2208 28297624

[4] Grimwade D Freeman SD Defining minimal residual disease in acute myeloid leukemia: which platforms are ready for “prime time”? Blood (2014) 124(23):3345–55. 10.1182/blood-2014-05-577593 25049280

[5] Lichtenegger FS Schnorfeil FM Hiddemann W Subklewe M Current strategies in immunotherapy for acute myeloid leukemia. Immunotherapy (2013) 5:63–78. 10.2217/imt.12.145 23256799

[6] Arpinati M Curti A. Immunotherapy in acute myeloid leukemia. Immunotherapy (2014) 6(1):95–106. 10.2217/imt.13.152 24341888

[7] Guillerey C Huntington ND Smyth MJ. Targeting natural killer cells in cancer immunotherapy. Nat Immunol (2016) 17(9):1025–36. 10.1038/ni.3518 27540992

[8] Vivier E Raulet DH Moretta A Caligiuri MA Zitvogel L Lanier LL Innate or adaptive immunity? The example of natural killer cells. Science (2011) 331(6013):44–9. 10.1126/science.1198687 21212348 PMC3089969

[9] Ljunggren HG Karre K In search of the ‘missing self’: MHC molecules and NK cell recognition. Immunol Today (1990) 11:237e44 10.1016/0167-5699(90)90097-S 2201309

[10] Parham P. Taking license with natural killer cell maturation and repertoire development. Immunol Rev (2006) 214:155e60. 10.1111/j.1600-065X.2006.00462.x 17100883

[11] Davis DM Chiu I Fassett M Cohen GB Mandelboim O Strominger JL. The human natural killer cell immune synapse. Proc Natl Acad Sci U S A (1999) 96:15062e7. 10.1073/pnas.96.26.15062 10611338 PMC24773

[12] Handgretinger R Lang P André MC. Exploitation of natural killer cells for the treatment of acute leukemia. Blood (2016) 127(26):3341–9. 10.1182/blood-2015-12-629055 27207791

[13] Moretta L Montaldo E Vacca P Del Zotto G Moretta F Merli P Human natural killer cells: origin, receptors, function, and clinical applications. Int Arch Allergy Immunol (2014) 164(4):253–64. 10.1159/000365632 25323661

[14] Ghiringhelli F Ménard C Martin F Zitvogel L. The role of regulatory T cells in the control of natural killer cells: relevance during tumor progression. Immunol Rev (2006) 214:229–38. 10.1111/j.1600-065X.2006.00445.x 17100888

[15] Ruggeri L Capanni M Casucci M Volpi I Tosti A Perruccio K Role of natural killer cell alloreactivity in HLA-mismatched hematopoietic stem cell transplantation. Blood (1999) 94(1):333–9. 10381530

[16] Ruggeri L Capanni M Urbani E Perruccio K Shlomchik WD Tosti A Effectiveness of donor natural killer cell alloreactivity in mismatched hematopoietic transplants. Science (2002) 295(5562):2097–100. 10.1126/science.1068440 11896281

[17] Ruggeri L Parisi S Urbani E Curti A. Alloreactive natural killer cells for the treatment of acute myeloid leukemia: from stem cell transplantation to adoptive immunotherapy. Front Immunol (2015) 6:479. 10.3389/fimmu.2015.00479 26528283 PMC4606119

[18] Locatelli F Moretta F Brescia L Merli P. Natural killer cells in the treatment of high-risk acute leukaemia. Semin Immunol (2014) 26(2):173–9. 10.1016/j.smim.2014.02.004 24613727

[19] Passweg JR Tichelli A Meyer-Monard S Heim D Stern M Kühne T Purified donor NK-lymphocyte infusion to consolidate engraftment after haploidenticalstem cell transplantation. Leukemia (2004) 18:1835–8. 10.1038/sj.leu.2403524 15457184

[20] Miller JS Soignier Y Panoskaltsis-Mortari A McNearney SA Yun GH Fautsch SK Successful adoptive transfer and in vivo expansion of human haploidentical NK cells in patients with cancer. Blood (2005) 105(8):3051–7. 10.1182/blood-2004-07-2974 15632206

[21] Rubnitz JE Inaba H Ribeiro RC Pounds S Rooney B Bell T NKAML: a pilot study to determine the safety and feasibility of haploidentical natural killer cell transplantation in childhood acute myeloid leukemia. J Clin Oncol (2010) 28(6):955–9. 10.1200/JCO.2009.24.4590 20085940 PMC2834435

[22] Curti A Ruggeri L D’Addio A Bontadini A Dan E Motta MR Successful transfer of alloreactive haploidentical KIR-ligand-mismatched natural killer cells after infusion in elderly high risk acute myeloid leukemia patients. Blood (2011) 118(12):3273–9. 10.1182/blood-2011-01-329508 21791425

[23] Curti A Ruggeri L Parisi S Bontadini A Dan E Motta MR Larger size of donor alloreactive NK Cell repertoire correlates with better response to NK cell immunotherapy in elderly acute myeloid leukemia patients. Clin Cancer Res (2016) 22(8):1914–21. 10.1158/1078-0432.CCR-15-1604 26787753

[24] Wagner J Pfannenstiel V Waldmann A Bergs JWJ Brill B Huenecke S A two-phase expansion protocol combining interleukin IL-15 and IL-21 improves natural killer cell proliferation and cytotoxicity against rhabdomyosarcoma. Front Immunol (2017) 8:676 10.3389/fimmu.2017.00676 28659917 PMC5466991

[25] Szmania S Lapteva N Garg T Greenway A Lingo J Nair B Ex vivo-expanded natural killer cells demonstrate robust proliferation in vivo in high-risk relapsed multiple myeloma patients. J Immunother (2015) 38(1):24–36. 10.1097/CJI.0000000000000059 25415285 PMC4352951

[26] Romee R Rosario M Berrien-Elliott MM Wagner JA Jewell BA Schappe T Cytokine-induced memory-like natural killer cells exhibit enhanced responses against myeloid leukemia. Sci Transl Med (2016) 8(357):357ra123. 10.1126/scitranslmed.aaf2341 27655849 PMC5436500

[27] Heidenreich S Kröger N. Reduction of relapse after unrelated donor stem cell transplantation by KIR-based graft selection. Front Immunol (2017) 8:41. 10.3389/fimmu.2017.00041 28228753 PMC5296332

[28] Bachanova V Cooley S Defor TE Verneris MR Zhang B McKenna DH Clearance of acute myeloid leukemia by haploidentical natural killer cells is improved using IL-2 diphtheria toxin fusion protein. Blood (2014) 123(25):3855–63. 10.1182/blood-2013-10-532531 24719405 PMC4064329

[29] Cooley S Trachtenberg E Bergemann TL Saeteurn K Klein J Le CT Donors with group B KIR haplotypes improve relapse-free survival after unrelated hematopoietic cell transplantation for acute myelogenous leukemia. Blood (2009) 113(3):726–32. 10.1182/blood-2008-07-171926 18945962 PMC2628378

[30] Boudreau JE Giglio F Gooley TA Stevenson PA Le Luduec JB Shaffer BC KIR3DL1/ HL A-B subtypes govern Acute Myelogenous Leukemia relapse after hematopoietic cell transplantation. J Clin Oncol (2017) 35(20):2268–78. 10.1200/JCO.2016.70.7059 28520526 PMC5501362

[31] Ghiringhelli F Ménard C Terme M Flament C Taieb J Chaput N CD4+CD25+ regulatory T cells inhibit natural killer cell functions in a transforming growth factor-beta-dependent manner. J Exp Med (2005) 202(8):1075–85. 10.1084/jem.20051511 16230475 PMC2213209

[32] Shimizu J Yamazaki S Sakaguchi SJ. Induction of tumor immunity by removing CD25+CD4+ T cells: a common basis between tumor immunity and autoimmunity. J Immunol (1999) 163(10):5211–8. 10553041

[33] Horton SJ Huntly BJ. Recent advances in acute myeloid leukemia stem cell biology. Haematologica (2012) 97(7):966–74. 10.3324/haematol.2011.054734 22511496 PMC3396664

[34] Khaznadar Z Boissel N Agaugué S Henry G Cheok M Vignon M Defective NK cells in acute myeloid leukemia patients at diagnosis are associated with blast transcriptional signatures of immune evasion. J Immunol (2015) 195(6):2580–90. 10.4049/jimmunol.1500262 26246143

[35] Costello RT Sivori S Marcenaro E Lafage-Pochitaloff M Mozziconacci MJ Reviron D Defective expression and function of natural killer cell-triggering receptors in patients with acute myeloid leukemia. Blood (2002) 99(10):3661–7. 10.1182/blood.V99.10.3661 11986221

[36] Fauriat C Just-Landi S Mallet F Arnoulet C Sainty D Olive D Deficient expression of NCR in NK cells from acute myeloid leukemia: evolution during leukemia treatment and impact of leukemia cells in NCRdull phenotype induction. Blood (2007) 109(1):323–30. 10.1182/blood-2005-08-027979 16940427

[37] Siegler U Kalberer CP Nowbakht P Sendelov S Meyer-Monard S Wodnar-Filipowicz A. Activated natural killer cells from patients with acute myeloid leukemia are cytotoxic against autologous leukemic blasts in NOD/SCID mice. Leukemia (2005) 19(12):2215–22. 10.1038/sj.leu.2403985 16224486

[38] Stringaris K Sekine T Khoder A Alsuliman A Razzaghi B Sargeant R Leukemia-induced phenotypic and functional defects in natural killer cells predict failure to achieve remission in acute myeloid leukemia. Haematologica (2014) 99(5):836–47. 10.3324/haematol.2013.087536 24488563 PMC4008119

[39] Curti A Trabanelli S Salvestrini V Baccarani M Lemoli RM. The role of indoleamine 2,3-dioxygenase in the induction of immune tolerance: focus on hematology. Blood (2009) 113(11):2394–401. 10.1182/blood-2008-07-144485 19023117

[40] Szczepanski MJ Szajnik M Welsh A Whiteside TL Boyiadzis M. Blast-derived microvesicles in sera from patients with acute myeloid leukemia suppress natural killer cell function via membrane-associated transforming growth factor-beta1. Haematologica (2011) 96(9):1302–9. 10.3324/haematol.2010.039743 21606166 PMC3166100

[41] Coles SJ Wang EC Man S Hills RK Burnett AK Tonks A CD200 expression suppresses natural killer cell function and directly inhibits patient anti-tumor response in acute myeloid leukemia. Leukemia (2011) 25(5):792–9. 10.1038/leu.2011.1 21274000 PMC3093357

[42] Baessler T Charton JE Schmiedel BJ Grünebach F Krusch M Wacker A CD137 ligand mediates opposite effects in human and mouse NK cells and impairs NK-cell reactivity against human acute myeloid leukemia cells. Blood (2010) 115(15):3058–69. 10.1182/blood-2009-06-227934 20008791

[43] Ayala F Dewar R Kieran M Kalluri R. Contribution of bone microenvironment to leukemogenesis and leukemia progression. Leukemia (2009) 23(12):2233–41. 10.1038/leu.2009.175 19727127 PMC4313556

[44] Isidori A Salvestrini V Ciciarello M Loscocco F Visani G Parisi S The role of the immunosuppressive microenvironment in acute myeloid leukemia development and treatment. Expert Rev Hematol (2014) 7(6):807–18. 10.1586/17474086.2014.958464 25227702

[45] Curti A Pandolfi S Aluigi M Isidori A Alessandrini I Chiodoni C Interleukin-12 production by leukemia-derived dendritic cells counteracts the inhibitory effect of leukemic microenvironment on T cells. Exp Hematol (2005) 33(12):1521–30. 10.1016/j.exphem.2005.08.005 16338495

[46] Mao Y Sarhan D Steven A Seliger B Lundqvist A Inhibition of tumor-derived prostaglandine-E2 blocks the induction of myeloid-derived suppressor cells and recovers natural killer cell activity. Clin Cancer Res (2014) 20(15):4096–106. 10.1158/1078-0432.CCR-14-0635 24907113

[47] Mantovani A Allavena P Sica A Balkwill F. Cancer-related inflammation. Nature (2008) 454:436–44. 10.1038/nature07205 18650914

[48] Gabrilovich DI Nagaraj S. Myeloid-derived suppressor cells as regulators of the immune system. Nat Rev Immunol (2009) 9:162–74. 10.1038/nri2506 19197294 PMC2828349

[49] Spaggiari GM Capobianco A Abdelrazik H Becchetti F Mingari MC Moretta L. Mesenchymal stem cells inhibit natural killer-cell proliferation, cytotoxicity, and cytokine production: role of indoleamine 2,3-dioxygenase and prostaglandin E2. Blood (2008) 111(3):1327–33. 10.1182/blood-2007-02-074997 17951526

[50] Cooley S Weisdorf DJ Guethlein LA Klein JP Wang T Le CT Donor selection for natural killer cell receptor genes leads to superior survival after unrelated transplantation for acute myelogenous leukemia. Blood (2010) 116:2411–9. 10.1182/blood-2010-05-283051 20581313 PMC2953880

[51] Mackinnon S Papadopoulos EB Carabasi MH Reich L Collins NH Boulad F Adoptive immunotherapy evaluating escalating doses of donor leukocytes for relapse of chronic myeloid leukemia after bone marrow transplantation: separation of graft-versus-leukemia responses from graft-versus-host disease. Blood (1995) 86(4):1261–8. 7632930

[52] Dazzi F Szydlo RM Craddock C Cross NC Kaeda J Chase A Comparison of single-dose and escalating-dose regimens of donor lymphocyte infusion for relapse after allografting for chronic myeloid leukemia. Cross NCP, Blood (2000) 95(1):67–71. 10607686

[53] Kiessling R Klein E Wigzell H “Natural” killer cells in the mouse. I. Cytotoxic cells with specificity for mouse Moloney leukemia cells. Specificity and distribution according to genotype. Eur J Immunol (1975) 5(2):112–7. 10.1002/eji.1830050208 1234049

[54] Kiessling R Klein E Pross H Wigzell H “Natural” killer cells in the mouse. II. Cytotoxic cells with specificity for mouse Moloney leukemia cells. Characteristics of the killer cell. Eur J Immunol (1975) 5(2):117–21. 10.1002/eji.1830050209 1086218

[55] Herberman RB Nunn ME Lavrin DH Natural cytotoxic reactivity of mouse lymphoid cells against syngeneic acid allogeneic tumors. I. Distribution of reactivity and specificity. Int J Cancer (1975) 16(2):216–29. 50294 10.1002/ijc.2910160204

[56] Siegler U Meyer-Monard S Jörger S Stern M Tichelli A Gratwohl A Good manufacturing practice-compliant cell sorting and large-scale expansion of single KIR-positive alloreactive human natural killer cells for multiple infusions to leukemia patients. Cytotherapy (2010) 12(6):750–63. 10.3109/14653241003786155 20491532

[57] Koehl U Kalberer C Spanholtz J Lee DA Miller JS Cooley S Advances in clinical NK cell studies: donor selection, manufacturing and quality control. Oncoimmunology (2015) 5(4):e1115178. 10.1080/2162402X.2015.1115178 27141397 PMC4839369

[58] Meyer-Monard S Passweg J Siegler U Kalberer C Koehl U Rovó A Clinical-grade purification of natural killer cells in haploidentical hematopoietic stem cell transplantation. Transfusion (2009) 49(2):362–71. 10.1111/j.1537-2995.2008.01969 19389215

[59] De Santis D Foley BA John E Senitzer D Christiansen FT Witt CS. Rapid, flow cytometric assay for NK alloreactivity reveals exceptions to rules governing alloreactivity. Biol Blood Marrow Transplant (2010) 16:179–91. 10.1016/j.bbmt.2009.10.026 19879950

[60] Fauriat C Andersson S Bjorklund AT Carlsten M Schaffer M Bjorkstrom NK Estimation of the size of the alloreactive NK cell repertoire: studies in individuals homozygous for the group A KIR haplotype. J Immunol (2008) 181:6010–9. 10.4049/jimmunol.181.9.6010 18941190

